# Cardiovascular progenitor cells and tissue plasticity are reduced in a myocardium affected by Becker muscular dystrophy

**DOI:** 10.1186/s13023-019-1257-4

**Published:** 2020-03-05

**Authors:** Martin Pesl, Sarka Jelinkova, Guido Caluori, Maria Holicka, Jan Krejci, Petr Nemec, Aneta Kohutova, Vita Zampachova, Petr Dvorak, Vladimir Rotrekl

**Affiliations:** 1 Department of Biology, Faculty of Medicine, Masaryk University, Kamenice 5, Brno, 62500 Czech Republic; 2 International Clinical Research Center, (ICRC), St. Anne’s University Hospital, Pekarska 53, Brno, 65691 Czech Republic; 3 1st Department of Cardiovascular Diseases, St. Anne’s University Hospital and Masaryk University, Pekarska 53, Brno, 65691 Czech Republic; 4 Central European Institute of Technology (CEITEC MU), Nanobiotechnology, Kamenice 5, Brno, 62500 Czech Republic; 5 Department of Cardiology, University Hospital Brno, Jihlavska 20, Brno, 62500 Czech Republic; 6 Center for Cardiovascular Surgery and Transplantation, Pekarska 53, Brno, 65691 Czech Republic; 7 1st Department of Pathology, Faculty of Medicine, Masaryk University and St. Anne’s University Hospital in Brno, Pekarska 53, Brno, 65691 Czech Republic

**Keywords:** Becker muscular dystrophy, Dystrophin, Cardiovascular progenitor cells, C-kit, Cardiomyopathy, Heart failure

## Abstract

**Abstract:**

We describe the association of Becker muscular dystrophy (BMD) derived heart failure with the impairment of tissue homeostasis and remodeling capabilities of the affected heart tissue. We report that BMD heart failure is associated with a significantly decreased number of cardiovascular progenitor cells, reduced cardiac fibroblast migration, and ex vivo survival.

**Background:**

Becker muscular dystrophy belongs to a class of genetically inherited dystrophin deficiencies. It affects male patients and results in progressive skeletal muscle degeneration and dilated cardiomyopathy leading to heart failure. It is a relatively mild form of dystrophin deficiency, which allows patients to be on a heart transplant list. In this unique situation, the explanted heart is a rare opportunity to study the degenerative process of dystrophin-deficient cardiac tissue. Heart tissue was excised, dissociated, and analyzed. The fractional content of c-kit^+^/CD45^−^ cardiovascular progenitor cells (CVPCs) and cardiac fibroblast migration were compared to control samples of atrial tissue. Control tissue was obtained from the hearts of healthy organ donor’s during heart transplantation procedures.

**Results:**

We report significantly decreased CVPCs (c-kit^+^/CD45^−^) throughout the heart tissue of a BMD patient, and reduced numbers of phase-bright cells presenting c-kit positivity in the dystrophin-deficient cultured explants. In addition, ex vivo CVPCs survival and cardiac fibroblasts migration were significantly reduced, suggesting reduced homeostatic support and irreversible tissue remodeling.

**Conclusions:**

Our findings associate genetically derived heart failure in a dystrophin-deficient patient with decreased c-kit^+^/CD45^−^ CVPCs and their resilience, possibly hinting at a lack of cardioprotective capability and/or reduced homeostatic support. This also correlates with reduced plasticity of the explanted cardiac tissue, related to the process of irreversible remodeling in the BMD patient’s heart.

## Background

Dystrophinopathies are a class of inherited X-linked genetic disorders that impair the proper synthesis of dystrophin, a scaffolding protein found in skeletal and cardiac muscle [1]. More than 70% of patients suffering from Becker muscular dystrophy (BMD), a mild form of dystrophin deficiency, are diagnosed with dilated cardiomyopathy (DCM) [2, 3], which is uncorrelated with skeletal muscle degeneration [4]. Heart failure is the most common cause of death in those with BMD [5], and patients are, in individual cases, referred for heart transplantation [6].

The cellular dynamics of DCM development in dystrophinopathies are still unclear. Although this condition involves mostly cardiomyocytes, increasing attention has been directed at understanding the features and progression of DCM in the non-muscular cell fractions. These mainly include cardiac fibroblasts [7–9] and endothelial cells [10–13], which are, in part, known to affect extracellular matrix and vascular remodeling. Other stromal cells possibly involved in cardiac homeostasis, survival, and disease dynamics are cardiovascular progenitor cells (CVPC) [14], which include the CD117^+^ (or c-kit)/CD45^−^ fraction, and has been the main focus of several recent studies [15, 16]. Currently, the evidence shows that c-kit^+^/CD45^−^ are activated by cardiac injury [17] supporting the hypothesis of paracrine regulation of cardiac function under pathophysiological conditions [16, 18, 19]; however, their role and fate in the human heart remains to be elucidated.

Here we describe the first comparative characterization of c-kit^+^/CD45^−^ CVPC occurrence and tissue plasticity in myocardium obtained during a rare case of a BMD heart transplant in our center, and only the third such transplantation in the Czech Republic during the past three decades of the transplantation program [20]. This unique sample from our BMD patient was compared to healthy control cardiac samples from healthy heart donors for cardiac transplantation.

## Results

### Patients’ history

A patient with muscular dystrophy developed mild disease-related symptoms at the age 9 yrs. and Gowers’ sign was pronounced by the age 15; additionally, there was calf pseudohypertrophy. To this day (age 48), the patient does not require a wheelchair and has no signs of intellectual impairment (for recent neurological report, see Additional file 1: Supplementary materials). Slowly progressing cardiomyopathy was detected in 2004 (age 33) and an internal cardiac defibrillator (ICD) was implanted in 2012 (the ICD was magnetic resonance incompatible, and no MRI scans could be performed). In 2014, the patient presented with heart failure. A cardiac evaluation found a reduced left ventricular ejection fraction (15%) with severe mitral regurgitation (3rd degree, hemodynamically graded as moderate for echo visualization; for a detailed description see Additional file 1: Supplementary materials and Additional file 2: Video 1; Additional file 3: Video 3; Additional file 4: Video 4), and borderline pulmonary hypertension, all of which responded to pharmacological intervention. Ischemic cardiomyopathy was excluded using coronary angiography (see Additional file 1: Supplementary materials). Due to progressive worsening of dyspnea up to the point of pulmonary edema, which required repeated hospital admissions, the patient was put on the non-urgent heart transplantation waiting list. The prescribed heart failure medication included beta-blockers, loop and mineralocorticoid diuretics, digoxin, angiotensin-converting enzyme (ACE) inhibitors, and proton pump inhibitors. A bicaval orthotropic cardiac transplantation took place shortly before the patient turned 44. He was discharged to out-patient care and follow-up 18 days post-transplantation. During the patient’s procedure and the five subsequent heart transplant procedures, surgically available left atrial (LA) tissue, from LA reductions (i.e., surgically required LA shaping and reductions in order to fit the transplant to the left atria of the recipient), were collected for use as healthy controls for this study.


**Additional file 2: Video 1.** Coronarography – right coronary artery.



**Additional file 3: Video 2.** Coronarography – left coronary artery.


**Fig. 1:**
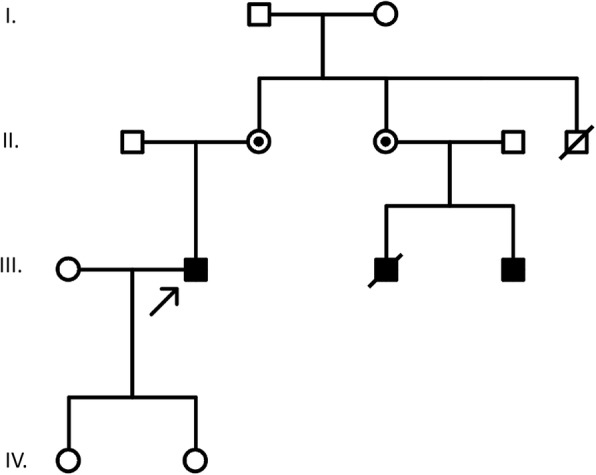
Family history and pedigree. Proband has no siblings, his father has no signs of muscular dystrophy nor cardiovascular disease and is 70 years old. Mother of patient is alive, with no cardiac involvement, genetic analysis was not performed. Mother’s sister, aunt of the proband, was diagnosed as a carrier of an identified mutation, has limited contact with the family, but no cardiac involvement could be traced. Her sons, proband’s first cousins, were both confirmed to carry the mutation. The older one (born 1971) died from heart failure in his early thirties, suffering frequent epileptic seizures, aggravating an already unfavorable status, with dystrophy signs since the age of 10. More detailed data could not be retrieved since he was more than 10 years deceased during manuscript preparation. The younger cousin, born 1982 is alive, in his late thirties, with severe myopathy, loss of ambulation since age 14 and was diagnosed with dilated cardiomyopathy. Mother’s brother died at 40 years, further details could not be retrieved from the family. The proband has two healthy daughters. Otherwise, the traceable family cardiac history was irrelevant

The patient had been screened for genetic mutations, in his twenties, i.e., exon sequencing of the *DMD* gene, but no known mutations were found. Due to the evident clinical presentation, other genes were not screened. A single point mutation c.3328 G > T, (p. Glu1110X) in exon 25, causing a stop codon, was later identified in a sample from the patient’s first-degree cousin, which was done as part of a family pedigree (see Fig. 1). The patient was subsequently diagnosed with the same mutation. This mutation would normally result in a DMD phenotype. Nevertheless the BMD phenotype of our patient, with a similar stop codon mutation in exon 25, was explained by the presence of alternatively spliced mRNA, in which a deletion bridges the non-sense mutation and thus partially suppresses its effect [21]. The patient has daughters, however, cascade mutation screening was not performed among female relatives, based on their preference. Creatine kinase (CK) levels before transplantation were only mildly increased, oscillating between 20 and 30 μkat/l (reference value under 3.17 μkat/l); the CK muscle brain (CK-MB) fraction was increased between 0.8–0.9 μkat/l, which normalized after transplantation (under 0.4 μkat/l). A skeletal muscle biopsy was not performed since the diagnosis was genetically verified.

### Gross pathologic description of BMD heart

The heart explant (size 150 × 120 × 70 mm) presented bilateral atrial and ventricular dilatation. Both coronary arteries showed sporadic fibrous and atheromatic plaques, without stenosis, and without perivascular inflammation. The wall thickness of the right ventricle (RV) was 4 mm along the anterior basal side and 3 mm along the posterior apical side. RV outflow was partially obstructed by a left ventricle (LV) septal mass. The ventricle walls presented with irregular fibrosis, a thinned or absent myocardial layer, and thickened or prevalent adipose tissue. The LV posterior wall, in particular, was irregularly thick, with the subvalvular wall being 5 mm with a non-compaction appearance (the non-compacted/spongiotic layer: compacted layer ratio was approximately 1). The middle part of the LV posterior wall had a thickness of 8 mm, with fibrosis and thinning toward the apex. The anterior LV wall was irregularly thick (5–10 mm) with focal fibrosis. The IVS thickness was 12 mm. A myxoid transformation of the tricuspid and mitral valves was observed. Histologically, there were partial and non-specific myocardial changes, i.e., dilated cardiomyopathy with hypertrophic cardiomyocytes and interstitial fibrosis. The free LV wall had foci of non-compaction, prominent adipose tissue, and small residual groups of cardiomyocytes. Similar changes were seen in the LV papillary muscles and the RV outflow tract. Laminar fibrosis was present mostly in the subepicardial and middle layers of the compact myocardium regions. Cardiomyocytes showed no signs of acute regressive changes, nor the pathologic changes typical of storage disorders. A slight focal intimal thickening with sporadic incipient atheromatic plaques was found in the coronary arteries.

**Fig. 2:**
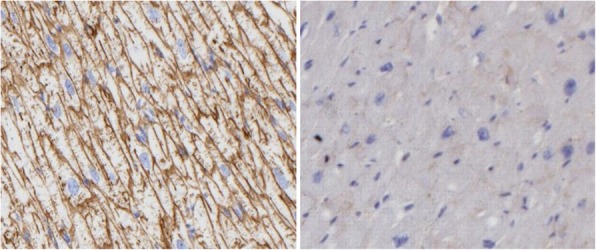
Immunohistopathology of human myocardium stained for presence of dystrophin. Left image: healthy ventricular myocardium showing membranous aggregation of dystrophin. Right image: Becker muscular dystrophy-affected ventricular myocardium, showing weak membranous distribution of dystrophin. Magnification 400X

BMD samples were compared with myocardial samples from hearts explanted due to ischemic heart disease. The controls showed more or less diffuse membranous dystrophin staining. The BMD patient’s samples showed a mosaic pattern with an absence of membranous staining and only sporadic isolated myocytes (< 0.1‰) with partial and weak membranous staining. These immuno-histopathological findings are shown in Fig. 2.

### C-kit^+^ /CD45^−^ cells are depleted in BMD-affected myocardium

**Fig. 3:**
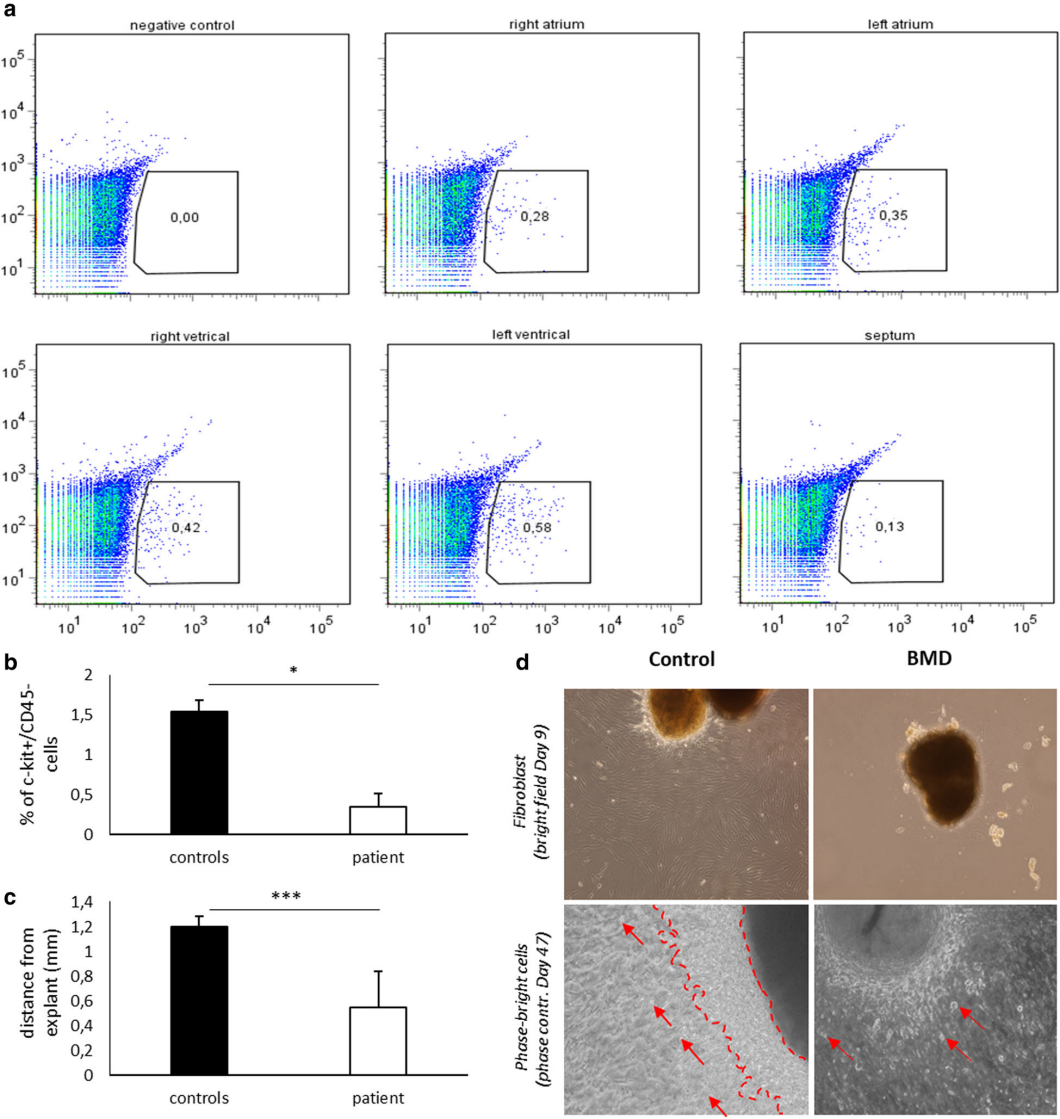
Results on the cardiac tissue analysis. **a** FACS analysis of c-kit^+^/CD45^−^ cells from dystrophin-deficient regions in different areas of the organ. **b** Comparison of c-kit^+^/CD45^−^ cells percentage fractions among negative controls (HD, left atrium) and the dystrophin-deficient heart (indicated parts), * = *p* < 0.05. **c** Maximum distance reached by fibroblasts at the end of the migration assay, statistical difference was assessed using Mann-Whitney test. (*** = *p* < 0.001, **d** Tissue sample images on an optical microscope: top row, bright field images show migrating fibroblasts from healthy samples, while few cells migrated from dystrophin-deficient samples; bottom row shows phase-contrast close-ups of the explants. HD fibroblast layers are covered by phase-bright cells (left, dashed area + red arrows), while dystrophin-deficient layers show only sparse migration of these cells (right, red arrows show examples)

We quantified the number of c-kit^+^/CD45^−^ cells to detect significant alterations in the cardiac c-kit^+^/CD45^−^ population fraction in heart tissue affected by BMD-DCM. Tissue samples from myocardium were dissociated, and single-cell suspensions were quantified with anti-CD117 (c-kit) and anti-CD45 fluorophore-conjugated antibodies. Tissue samples were excised from the whole myocardium of the explanted BMD heart (i.e., LA, right atrium (RA), LV, RV, and intraventricular septum (IVS)) and compared to LA samples of healthy heart donors (HD). While the HD atrial samples contained an average of 1.54% (1.39; 1.62, and 1.62% for each HD sample) c-kit^+^/CD45^−^ cells, samples from the BMD myocardium contained 0.28 and 0.35% c-kit^+^/CD45^−^ cells (RA and LA, respectively; see Fig. 3a top row), which was less than a quarter of the HD average. The BMD ventricular samples contained 0.42, 0.58, and 0.13% c-kit^+^ /CD45^−^ cells (RV, LV, and IVS, respectively; see Fig. 3a bottom row). The BMD c-kit^+^/CD45^−^ mean fraction was significantly lower with respect to the mean value of the HD atrial samples (*p* = 0.036, calculated using the Mann-Whitney statistical test, Fig. 3b).

### BMD heart tissue shows ex vivo reduced plasticity and phase-bright cells resilience

We evaluated the ability of mildly dissociated tissue explants (< 1 mm^3^ pieces) to adhere to culture dishes, in order to prove their ability to retain tissue plasticity. While BMD explants samples required 7 days of ex-vivo cultivation before starting to attach, the HD samples started to attach within 4 days. We then observed and evaluated cardiac fibroblast cell migration from 19 HD and 13 BMD explants-derived samples to estimate the remodeling capacity of the original tissue. The maximal migration distance of fibroblasts from the explant border after 9 days in vitro was 1031.2 ± 351.2 μm in HD samples, but only 330.9 ± 224.4 μm in BMD (*p* < 0.001, Mann-Whitney, Fig. 3c and d top row). We later observed phase-bright cells on fibroblasts. Phase-bright cells have been previously reported to contain a c-kit^+^ fraction [22]. These loosely adherent cells were thus stained for the presence of the c-kit marker, which revealed a 61 ± 20% positive fraction (6 explant samples analyzed, see Additional file 1: Supplementary materials). Phase-bright cells in HD samples started to appear on the fibroblast layer after 9 days in vitro. In contrast, phase-bright cells in BMD samples only appeared on fibroblasts after 28 days. BMD explants showed few isolated phase-bright cells on the surrounding fibroblast layer, while HD sample had a dense covering on the surrounding explant tissue (Fig. 3D bottom row). Overall, 32.45% (37/114) of the HD explants showed phase-bright cells, while only 15.64% (23/147) of BMD samples showed phase-bright cells. After 90 days, the HD phase-bright cells had grown enough to allow immunolabelling. The BMD phase-bright cells started to disappear before reaching the quantity necessary for immunolabeling and were no longer observable after 51 days of cultivation, which impaired the comparative analysis.

## Discussion

Heart failure due to DCM is a common clinical manifestation in patients with BMD [5]. It has been shown that CVPCs, including c-kit^+^/CD45^−^ cells, are involved in cardiac injury and degeneration [17, 23], resulting, for example, in capillary rarefaction, fibrosis, and other pathogeneses of an affected heart [24, 25]. The pathogenesis of dystrophinopathy was previously linked to increased DNA damage and to increased mutagenesis in pluripotent stem cells reprogrammed from DMD patients and lacking dystrophin, as we showed previously [26]. This could affect different organ tissue stem cells or niche supporting cells similar to satellite cells [27]. In this study, we investigated the presence of CVPCs in one model BMD-affected myocardium, as well as tissue remodeling capabilities. We observed that the CVPC fraction found in the dystrophic LA was more than four times lower than the average found in HD samples. The detected values of BMD c-kit^+^/CD45^−^ fractions were also comparable to previously reported numbers in idiopathic DCM [28]. This CVPCs depletion resembles DCM associated with accelerated aging [29] and can be caused by an increased turnover concomitant with cell damage. These mechanisms of depletion have been shown, for instance, in hematopoietic stem cells with mutated Sirt1 [30], or dystrophin-deficient ckit^+^/CD45^−^ in the GRMD dog model [31]. The reduced tissue plasticity in our model, observed through decreased adherence and cell migration from diseased tissue, also might point to impaired remodeling properties of the BMD-DCM heart. In concert with this hypothesis, only one-tenth of the BMD tissue fragments adhered to gelatin-coated culture dishes, which was almost three-times less than for HD tissue. Dystrophic samples also required nearly twice as much time to attach. Decreased culture dish adherence of BMD heart explanted tissue was accompanied by reduced migration ability of the outgrowing cells. Such behavior could originate in the presence of an activated myofibroblast subpopulation, due to a chronic state of myocardial injury and inflammation [32] with progressive fibrosis [33, 34]. Myofibroblasts are a specialized cell type in between the phenotype of cardiac fibroblasts and smooth muscle myocytes that express α-smooth muscle actinin (SMA) and play an important role in cardiac remodeling [35, 36]. Their motility is reduced due to overexpression of focal adhesion proteins [37] and reduced production of collagen VIII, the lack of which has been associated with decreased migratory abilities [38]. Thus, the presence of terminally differentiated myofibroblasts might explain the reduced adhesion and migration of the cells in vitro.

Ultimately, impaired survival rate of phase-bright cells in BMD samples was observed, while these cells appeared and persisted in the HD fibroblast layer. Moreover, in the BMD samples they were only sparsely identifiable and prematurely disappeared from the BMD fibroblast layer. Their limited in vitro survival in dystrophin-deficient heart explants might hint at altered c-kit^+^/CD45^−^ cell resilience also in vitro and subsequently could promote BMD-DCM progression.

The main limitation of the study is the unique characteristic of the human BMD heart sample since only one specimen was available and opportunities to study a heart affected by dystrophinopathy are extremely rare. A comparable sampling of healthy human ventricular tissue was impossible and clearly unethical; thus, atrial tissue controls came from transplants, considered healthy from a cardiac point of view and thus suitable for heart transplantation. We chose donors of similar age to the BMD patient to remove the age bias; with one exception, all were males. Still, tissue availability was limited with only minimal information regarding the medical backgrounds of the donors and families.

In non-ischemic DCM, LA remodeling was observed in functional and morphological changes [39], making this heart area a valid control source. The wide age interval of the donor cohort (see Additional file 1: Supplementary materials) is common in other similar studies [28], and was not significantly different from the age of the BMD patient, as shown using the one-sample t-test (*p* = 0.625). To the best knowledge of the authors, this analysis is the first to directly quantify c-kit^+^/CD45^−^ cells from BMD-DCM heart tissue. It contributes to the present understanding of the occurrence and role of c-kit^+^/CD45^−^ cells by showing a comparative reduction in this cell fraction, which can be correlated with reduced plasticity and cellular resilience in the BMD heart.

## Conclusions

Our findings give further insights into the correlation between c-kit^+^/CD45^−^ CVPCs levels, irreversible remodeling, and heart failure in BMD. Future studies aimed at describing c-kit^+^/CD45^−^ mechanisms of activation, paracrine modulation, and genetic impairment can provide new therapeutic perspectives into the management of cardiomyopathies. The analysis of c-kit^+^/CD45^−^ cells or possibly their secretome might serve as a helpful biomarker for early stages of cardiomyopathy, possibly not limited only to BMD patients.

## Methods

### Flow cytometry assay

Tissue samples were excised (in BMD) or extracted during a left atrial biopsy (from heart donors HD). Reduced dystrophin expression was confirmed by immunohistochemistry of myocardial samples. BMD samples were taken from different sections of the heart (left and right atrium, left and right ventricle, and the interventricular septum). As for the healthy donors, only adult patients were included (age 24–62 years, variance statistically insignificant *p* = 0.625) to exclude the bias of pediatric patients that have increased CVPC levels [23]. Due to the unavailability of ventricular samples, only atrial samples, which were obtained from surgical reduction before placing the healthy heart into the chest cavity during transplantations, were used. No other differences in CVPC levels were reported. Samples were treated as previously described by Messina et al. [22]. Briefly, tissue samples were immersed in a 60 mm Petri dish (TPP, Trasadingen, CH) filled with 1xPBS + 2% Pen/Strep (BioSera, Nuaille, France) and cut into 1–2 mm^3^ pieces with scissors. Tissue fragments were digested in a dissociating solution composed of 0.1% Collagenase IV in DMEM-F12 (both from Life Technologies, Carlsbad, CA, USA). Dissociation was carried out for 2 h at 37 °C with gentle manual agitation every 15 mins. The dissociation was stopped by adding an equal volume of explant IMDM medium (Life Technologies) supplemented with FBS 10% (Life Technologies), β mercaptoethanol 1% (Sigma-Aldrich, St. Louis, MO), Pen/Strep 1%, and L-glutamine 1% (both from BioSera). Samples were collected and pipetted vigorously with a 1 ml tip to release cells from tissue blocks. The dissociated cell suspension was filtered through a 40 μm strainer (Costar, Washington DC, USA) and spun in a conical tube at 200×g for 5 min. The supernatant was removed, and the cell pellet was resuspended in FACS-PBS, made from 1xPBS with 0.5% BSA (Sigma Aldrich) and 2 mM EDTA (Penta, Prague, CZ). Spinning and resuspension were repeated 3 times to ensure enzyme removal and cell separation. Antibodies were finally added to the suspension (CD45-FITC, cl. 30F11; CD117-APC, cl.3C11, all from Miltenyi Biotech, Bergisch Gladbach, DE) and the analysis was run on a BD FACS Canto II (BD Biosciences, New Jersey, NJ-USA). The negative control for FACS was an unlabeled cell suspension. Data were analyzed using FlowJo (FlowJo LLC, Ashland, OR-USA).

### Cell migration assay and immunocytochemistry

Samples were cut into 1–2 mm^3^ pieces, as described in the previous paragraph. For the migration assay and c-kit^+^/CD45^−^ cell cultivation, the tissue explants were collected in a dissociation solution and incubated for 5 mins at 37 °C. The enzymatic solution was then removed and replaced with a fresh solution. Incubation and enzyme replacement was repeated three times. Dissociation was stopped by adding an equal volume of explant medium. The samples were spun at 200×g for 2 min, then resuspended with explant medium. The tissue explants were distributed evenly on a 0.1% gelatin-treated 6-well plates (TPP), in 5 ml of explant medium. The dishes were not moved for 4 days to allow attachment of the tissue. The medium was changed every 4 days, and photos were taken on the same days using an inverted Olympus IX71 microscope (Tokyo, Japan), and QuickPhoto Camera software (Promicra, Prague, CZ). Pictures were analyzed using ImageJ open-source software to measure migration [40] distance and phase-bright cells. C-kit+ cells were detected using CD117-APC (Miltenyi Biotec, Bergisch Gladbach, DE) dissolved in 1:100 proportion in IMDM + 0.5% BSA. Photos were taken using a Zeiss LSM700 confocal laser scanning microscope (Zeiss, Oberkochen, DE).

### Histopathological methods

The heart explants were dissected according to standard procedures, samples were taken from right atrium, tricuspid valve; the anterior and posterior wall, and outflow tract of the right ventricle; the right coronary artery; the right atrium and mitral valve; anterior papillary muscles; multiple samples from the anterior and posterior wall of the left ventricle; the septum and left coronary arteries. For immune-histological identification of dystrophin, formalin-fixed, paraffin-embedded tissue sections were treated with heat antigen retrieval (95 °C for 45 min, pH 9), and then treated with primary antibodies (Dystrophin Mouse Monoclonal Antibody, Leica, clone 34C5) using the avidin-biotin-immunoperoxidase method according to the manufacturer’s protocol.

### Statistical analysis

Descriptive statistics and comparisons between sample groups were performed using Prism 5.0 software (GraphPad, La Jolla, CA-US). Available normality tests were performed, and the non-parametric Mann-Whitney test was used to estimate statistical differences between samples. A *p*-value less than 0.05 was considered significant.

## Supplementary information

**Additional file 1.** Supplementary materials.

**Additional file 4: Video 3.** Echocardiography.

## Data Availability

The data that support the findings of this study are available from the corresponding author V.R. upon reasonable request.

## Abbreviations

BMD: Becker muscular dystrophy
DCM: Dilated cardiomyopathy
HTx: Heart transplantation
IVS: Interventricular septum
LV: Left ventricle
PBC: Phase-bright cells
RV: Right ventricle
