# Supplementary material for: Cardiovascular progenitor cells and tissue plasticity are reduced in a myocardium affected by Becker muscular dystrophy
Source: Orphanet J Rare Dis. 2020 Mar 5;15:65. doi: 10.1186/s13023-019-1257-4 (PMC7057505; doi:10.1186/s13023-019-1257-4)
Supplement: Supplementary file 3 — Additional file 1. Supplementary materials. [file 13023_2019_1257_MOESM1_ESM.docx]

# **Cardiovascular progenitor cells and tissue plasticity are reduced in a myocardium affected by Becker muscular dystrophy**

# **Supplementary materials**

*Neurological findings provided by attending neurologist with patient consent in time of transplantation*

Full MRC scaling was not performed on the initial visits and was only partially implemented later, during follow-up. There was no cognitive impairment, cranial nerves examination is without abnormalities. Upper extremities able to elevate against gravity only (Grade 2+). There were no contractures, rather pronounced hypermobility. Lower extremities findings include m. psoas force 4-, quadriceps 4+, and calf hypertrophy.

The patient was able to stand on the tiptoes, unable to stand on heels, there was generalized areflexia, pronounced Gower’s sign, able to stand up from chair using “trick” movements. The head raise and holding was normal, no scoliosis. There was an accentuation of lumbar lordosis.

*Echocardiography findings before transplantation*

The patient presented a dilated left ventricle with a spheroid shape, systolic diameter 70 mm and diastolic 77 mm, with severely impaired systolic function, without regionally specific kinetic impairment. The global ejection fraction was 15% with slightly asynchronous contraction. There is no pericardial effusion, nor any pathological intracardiac masses. The septum and posterior wall both had a thickness of 8 mm. The right ventricle had a diastolic dimension of 32 mm and a ICD electrode was present in the right heart chambers. The mitral valve showed 3^rd^ degree regurgitation and was hemodynamically moderate, other valves did not show hemodynamically important findings. The tricuspid valve showed a gradient of 23-25 mmHg and the estimated right ventricular pressure was 40 mmHg. TAPSE was 22, the inferior vena cava was 24 mm and collapsed partially during respiration. The left atrium has 47 mm with grade II diastolic dysfunction and an elevated left atrial filling pressure. The ACT was 135 ms.

*Healthy sample donors*

Healthy ventricular tissue was unavailable for sampling for clear ethical and common-sense reasons. Healthy atrial tissue can be sampled during transplantations performed using the bicaval technique. During operations, the graft atria must be reduced to fit remaining part of acceptor atria and thus surplus tissue can be utilized for analyzes. In addition to the BMD patient’s whole heart samples, atrial samples from six transplantations were sampled and analyzed. The donors’ clinical characteristics are listed in Table 1. The average donor age was 47.5 ± 11.4 years. A single female heart was among the six analyzed. All the samples were acquired under sterile conditions and kept in explant medium up to 1 hour until preparation.

Table 1: Summary of the cardiovascular history of hTX donors and analysis performed.

| Age | Sex | Cardiovascular history | Analysis |
| --- | --- | --- | --- |
| 42 | male | no cardiovascular history, ejection fraction 60, smooth coronaries | FACS/migration |
| 56 | male | no cardiovascular history, ejection fraction 65, smooth coronaries | FACS |
| 52 | female | hypertension controlled on medication, ejection fraction 63, coronaries up to 20% reduction of lumen | FACS |
| 45 | male | hypertension controlled on medication, ejection fraction 64, coronaries up to 10% reduction of lumen | migration |
| 24 | male | no cardiovascular history, ejection fraction 65, smooth coronaries | migration |
| 62 | male | recently diagnosed hypertension uncontrolled on setting up medication, ejection fraction 60, smooth coronaries | migration |

## **Supplementary results**

### *Individual findings*


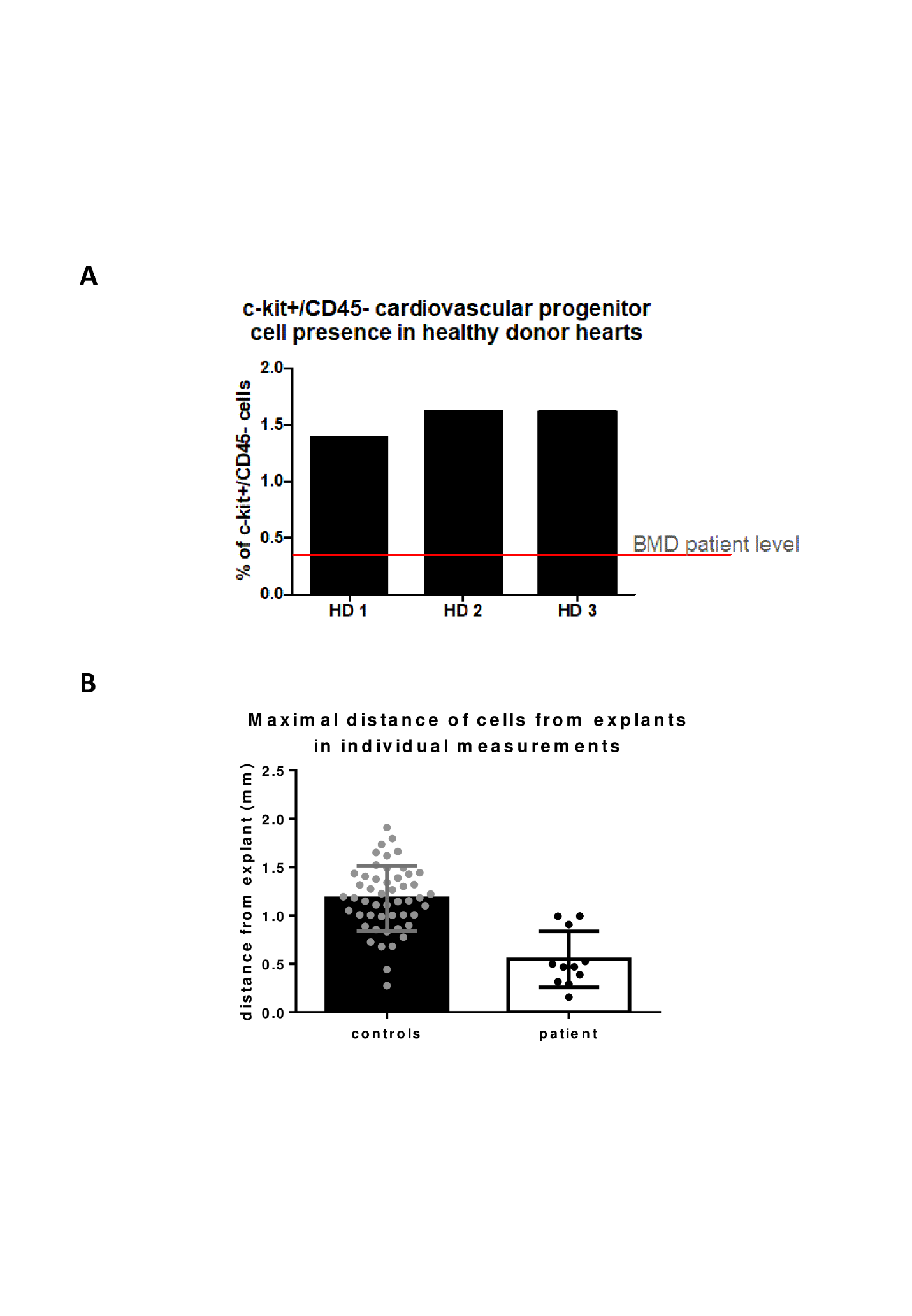
*Supplementary Figure 1: (A) Individual cell count for ckit+/CD45- CPVCs across the healthy atrial samples (coefficient of variation 8.6%). The red line represents the CVPC average level in the BMD patient. (B) Individual distances of 4 WT patients’ cells with at least 9 images evaluated (coefficient of variation 28.5%) and 11 images were evaluated from the BMD patient samples from various (mostly LA and RV) sections of the heart (coefficient of variation 53.0%).*

### *Immunocytochemistry*


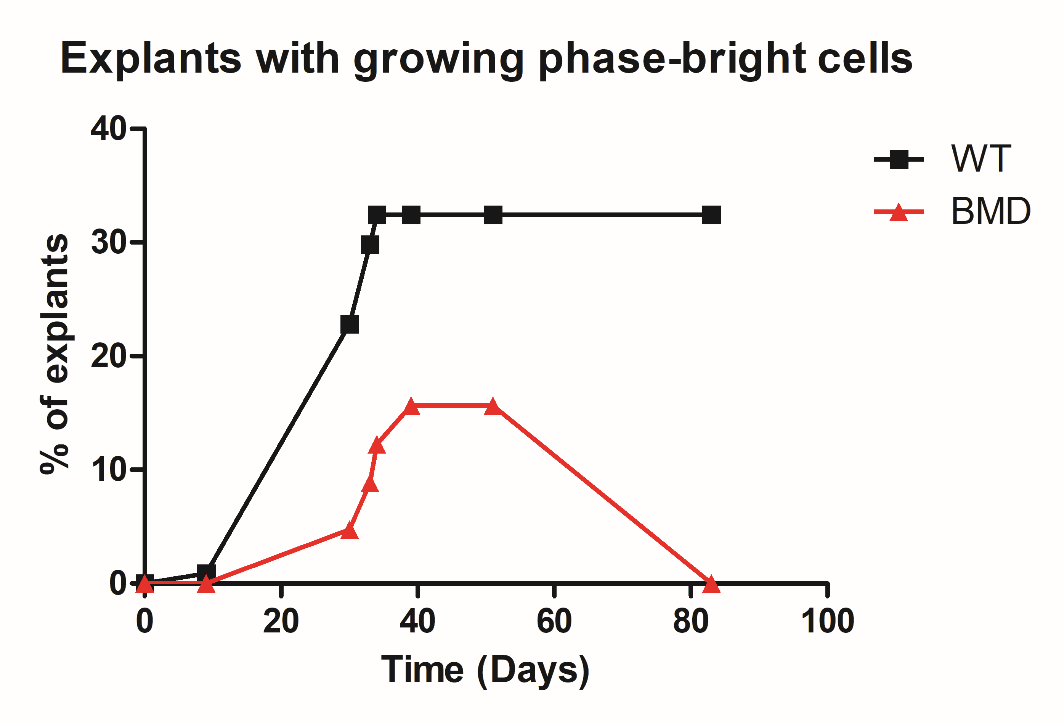
Healthy explanted tissue samples were cultivated for 3 months (94 days) while first shedding adherent fibroblasts and later the loosely adherent phase-bright cells. These cells were less pronounced on BMD samples, in smaller numbers, and with decreased survival rates (Supplementary Fig. 1). To establish the presence of c-kit^+^ cells, the samples were incubated with CD117 (c-kit) antibody, which showed positive labeling in 61 ± 20% of these phase-bright cells (Supplementary Fig. 2). A similar analysis was not possible for the dystrophic samples due to low numbers and early loss of these phase-bright cells.

*Supplementary Figure 2: Time graph of the percentage of explants presenting phase-bright cells between healthy samples (WT) and affected by Becker Muscular Dystrophy (BMD)*


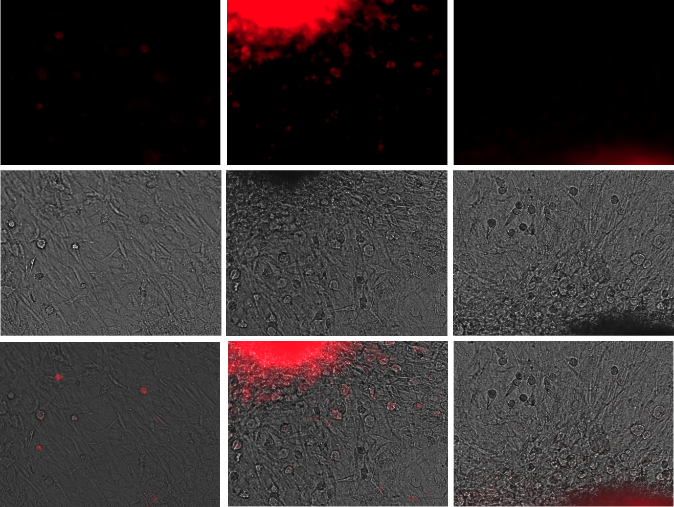


c-kit

bright field

merged

**negative**

**labeled healthy explants**

*Supplementary Figure 3: Labeling of the c-kit marker in the explanted tissue samples of a healthy heart (two first columns) and compared to no signal in the non-stained sample (right column), which shows the presence of c-kit+ populations among the phase-bright outgrown cells. Bar represents 100 µm.*

**References**

[1] E. Messina, L. De Angelis, G. Frati, S. Morrone, S. Chimenti, F. Fiordaliso, M. Salio, M. Battaglia, M.V.G. Latronico, M. Coletta, E. Vivarelli, L. Frati, G. Cossu, A. Giacomello, Isolation and expansion of adult cardiac stem cells from human and murine heart, Circ. Res. 95 (2004) 911–921. doi:10.1161/01.RES.0000147315.71699.51.

[2] K.K. Treloar, M.J. Simpson, Sensitivity of Edge Detection Methods for Quantifying Cell Migration Assays, PLOS ONE. 8 (2013) e67389. doi:10.1371/journal.pone.0067389.
